# Type 1 Diabetes Mellitus in the SARS-CoV-2 Pandemic: Oxidative Stress as a Major Pathophysiological Mechanism Linked to Adverse Clinical Outcomes

**DOI:** 10.3390/antiox10050752

**Published:** 2021-05-09

**Authors:** Aikaterini Kountouri, Emmanouil Korakas, Ignatios Ikonomidis, Athanasios Raptis, Nikolaos Tentolouris, George Dimitriadis, Vaia Lambadiari

**Affiliations:** 1 Second Department of Internal Medicine, Attikon University Hospital, Medical School, National and Kapodistrian University of Athens, 12462 Athens, Greece; KaterinaK90@hotmail.com (A.K.); mankor-th@hotmail.com (E.K.); atraptis@med.uoa.gr (A.R.); 2 Second Cardiology Department, Attikon University Hospital, Medical School, National and Kapodistrian University of Athens, 12462 Athens, Greece; ignoik@gmail.com; 3 First Department of Propaedeutic and Internal Medicine, Medical School, National and Kapodistrian University of Athens, Laiko General Hospital, 11527 Athens, Greece; ntentolouris@yahoo.gr; 4 Sector of Medicine, Medical School, National and Kapodistrian University of Athens, 11527 Athens, Greece; gdimitr@med.uoa.gr

**Keywords:** inflammation, immunity, oxidative stress, SARS-CoV-2, type 1 diabetes mellitus, hyperglycemia, endothelial dysfunction, ACE2

## Abstract

Recent reports have demonstrated the association between type 1 diabetes mellitus (T1DM) and increased morbidity and mortality rates during coronavirus disease (COVID-19) infection, setting a priority of these patients for vaccination. Impaired innate and adaptive immunity observed in T1DM seem to play a major role. Severe, life-threatening COVID-19 disease is characterized by the excessive release of pro-inflammatory cytokines, known as a “cytokine storm”. Patients with T1DM present elevated levels of cytokines including interleukin-1a (IL), IL-1β, IL-2, IL-6 and tumor necrosis factor alpha (TNF-α), suggesting the pre-existence of chronic inflammation, which, in turn, has been considered the major risk factor of adverse COVID-19 outcomes in many cohorts. Even more importantly, oxidative stress is a key player in COVID-19 pathogenesis and determines disease severity. It is well-known that extreme glucose excursions, the prominent feature of T1DM, are a potent mediator of oxidative stress through several pathways including the activation of protein kinase C (PKC) and the increased production of advanced glycation end products (AGEs). Additionally, chronic endothelial dysfunction and the hypercoagulant state observed in T1DM, in combination with the direct damage of endothelial cells by severe acute respiratory syndrome coronavirus 2 (SARS-CoV-2), may result in endothelial and microcirculation impairment, which contribute to the pathogenesis of acute respiratory syndrome and multi-organ failure. The binding of SARS-CoV-2 to angiotensin converting enzyme 2 (ACE2) receptors in pancreatic b-cells permits the direct destruction of b-cells, which contributes to the development of new-onset diabetes and the induction of diabetic ketoacidosis (DKA) in patients with T1DM. Large clinical studies are required to clarify the exact pathways through which T1DM results in worse COVID-19 outcomes.

## 1. Introduction

Coronavirus disease (COVID-19) is a newly recognized infection that was first reported in Wuhan, China in December 2019 and has rapidly spread worldwide. COVID-19 is caused by a novel enveloped RNA beta-coronavirus named severe acute respiratory syndrome coronavirus 2 (SARS-CoV-2) [1,2]. On 11 March 2020, the World Health Organization (WHO) declared the outbreak of COVID-19 as a pandemic after recording 118,000 cases globally in 114 different countries [3]. The clinical presentation of COVID-19 infection ranges from no or mild symptoms to critical illness including multi-organ failure and even death [4,5]. The mortality and morbidity rates of COVID-19 infection are increased in patients with underlying comorbidities [6]. Several epidemiological studies have pointed out that the percentage of underlying comorbidities such as diabetes, hypertension and cardiovascular diseases was significantly higher in COVID-19 patients requiring hospitalization in a medicine or intensive care unit [7,8,9]. Regarding diabetes in particular, the Center for Disease Control and Prevention reports that patients with diabetes are more prone to severe illness and to poorer clinical outcomes [10].

In the literature there are several reports regarding the prevalence, the clinical outcomes, and the pathophysiological mechanisms of COVID-19 in patients with type 2 diabetes mellitus (T2DM) [11,12,13]. However, research data concerning the effect of SARS-CoV-2 on patients with type 1 diabetes mellitus (T1DM) are scarce [14]. A multicenter study from the United Kingdom was the first that investigated the characteristics and clinical outcomes of COVID-19 infection in patients with T1DM, reporting that severity and mortality risk are also higher in T1DM. Furthermore, research data demonstrated that hyperglycemia is the most prevalent presenting symptom of COVID-19 disease, and diabetic ketoacidosis (DKA) is the most frequent adverse outcome in patients with preexisting T1DM [15]. Inversely, the preceding hyperglycemia in patients with T1DM is significantly associated with COVID-19-related mortality [16]. Interestingly, ambient hyperglycemia has been reported as an independent predictor for mortality and morbidity in patients with severe acute respiratory syndrome [17]. Moreover, SARS-CoV-2 has been suggested as a potential inducer of new-onset T1DM [18]. Based on the aforementioned research data, several potential factors and pathophysiological mechanisms, alone or in combination, may increase the susceptibility of subjects with T1DM to serious illness following COVID-19 infection. However, data about these mechanisms are scarce. The aim of the review is to describe the possible pathophysiological pathways that are associated with worse COVID-19 outcomes specifically in patients with T1DM.

## 2. Material and Methods

The studies mentioned in this review were retrieved by a computer search program using the PubMed, Scopus and Web of Science electronic databases. The authors searched for scientific literature published in English up to February 2021. The applied search terms were a combination of the following: “coronavirus”, “COVID-19”, “SARS-CoV2”, “ACE2”, “type 1 diabetes mellitus”, “hyperglycemia”, “endothelium”, “inflammation”, “oxidative stress”, “coagulopathy” and “immunity”. Additional references were identified from reviewing the references cited in the original articles. The final reference list was generated on the basis of relevance to the topics covered in this publication, with the aim of covering multiple aspects of the association between T1DM and COVID-19, and describing possible pathophysiological mechanisms leading to adverse clinical outcomes in patients with T1DM during COVID-19 infection.

## 3. T1DM and COVID-19 Disease: Epidemiological Data

Diabetes—especially when poorly controlled—has been recognized as a strong and independent predictor of severe outcomes including death during COVID-19 infection [19,20,21,22,23]. In a systematic review of the literature, Apicella et al. [24] summarized the potential risk factors that could lead to worst outcomes in COVID-19-positive subjects with diabetes: hyperglycemia, older age, male sex, non-white ethnic groups, poor socioeconomic status, comorbidities (such as hypertension, cardiovascular/cerebrovascular disease, chronic kidney disease), obesity, inflammation, and coagulation [24]. However, although it can be assumed that most of the patients reported in the literature with severe outcomes from SARS-CoV2 had type 2 diabetes (T2DM), the information concerning type 1 diabetes (T1DM) is still scarce.

The risk for severe COVID-19 disease in patients with T1DM has been investigated in the following studies: (a) Ebekozien et al. [15] reported clinical outcomes in 33 COVID-19-positive subjects with T1DM—mean age was 24.8 years, median HbA1c was 8.5%, obesity/hypertension/cardiovascular disease were the most prevalent comorbidities and diabetic ketoacidosis the most prevalent adverse outcome (45.5% of the cases). (b) Two reports by Barron et al. [25] and Holman et al. [14] from NHS UK covering almost all the general practices in England were the first to compare the risk for severe outcomes between T1DM and T2DM during COVID-19 infection addressing all the potential risk factors, including previous glycemic control. The results (adjusted for confounding factors) demonstrated that 1/3 of all in-hospital deaths with COVID-19 occurred in people with diabetes; the overall risk was 3.5 times higher in those with T1DM, while people with T2DM were at about twice the risk, compared to people without a known diagnosis of diabetes. In people with T1DM, preceding hyperglycemia was significantly associated with COVID-19-related mortality at values of HbA1c >10%, whereas in those with T2DM this risk was significant at HbA1c of 7.6% or higher. Obesity (body mass index (BMI) > 30 kg/m^2^) was almost equally associated with COVID-19-related deaths in both diabetes types. Old age (>70 years), male sex, impaired renal function, previous cardiovascular comorbidities, deprived people in under-privileged communities and people of black ethnicity were also significantly associated with the highest risk of in-hospital death from COVID-19. These results are supported by a previous study in over 100,000 primary care adult patients with diabetes in England: those with T1DM and T2DM had ~2-3-fold higher rates of all infections (including pneumonia, sepsis, and endocarditis) compared to patients without diabetes. A comparison of T1DM and T2DM revealed that the risk of hospitalization and infection-related death in the former versus the latter was 2-fold and 4-fold higher, respectively; older age (≥70 years), morbid obesity (BMI > 40 kg/m^2^), serious comorbidities (cardiovascular disease, hypertension, chronic kidney failure) and residence in more deprived areas markedly increased the risk for severe infections and adverse outcomes, whereas the duration of diabetes seemed to play a role only in patients with T2DM [26]. (c) McGurnaghan et al. [27] investigated the risk factors for severe outcomes in COVID-19-infected subjects with T1DM and T2DM in the total population of Scotland. The authors showed that subjects with diabetes had ~40% greater risk of developing fatal or critical care unit-treated COVID-19 disease compared to people without diabetes after adjustment for age and sex; the risk was ~1.75 times higher in T1DM than in T2DM. Male sex, residence in deprived areas, comorbidities (retinopathy, chronic kidney disease), longer diabetes duration, worse glycemic control and previous hospitalization for hypoglycemia or ketoacidosis in the past five years were among the most prevalent risk factors. Adjustment for age, sex and diabetes duration revealed that people with T1DM and T2DM had a similar risk for COVID-19-related severe outcomes [27]. (d) In a prospective cohort study, Gregory et al. [28] identified 40 COVID-19-positive patients with T1DM across a regional healthcare network of 137 service locations using electronic health records at Vanderbilt University Medical Center. The risk for severe illness and hospitalization in T1DM (BMI ~25 kg/m^2^) was 3- to 4-fold higher than in patients without diabetes and similar to that in T2DM. COVID-19 outcome severity in T1DM was related to poor glycemic control within the past year (HbA1c ~8%), the presence of chronic complications (hypertension, retinopathy, chronic kidney disease, neuropathy), older age, black ethnicity and low socioeconomic status [29]. (e) In a recent study, Ebekozien et al. [30] investigated the prevalence of ketoacidosis among 180 non-Hispanic white, non-Hispanic black and Hispanic subjects with T1DM during COVID-19 infection in 52 clinical sites across the USA. The results showed that non-Hispanic blacks were more prone to develop ketoacidosis, suggesting the role of race and ethnicity. In contrast to non-Hispanic blacks, most of the non-Hispanic white patients were using insulin pumps and continuous glucose monitoring systems (CGMS) in their treatment. (f) In contrast to these reports, Vangoitsenhoven et al. [30] studied the hospitalization needs of 2336 subjects with T1DM from two diabetes specialist centers in the community during the first 3 months of the pandemic in Belgium. Of this cohort, only 0.21% were admitted to the hospital for treatment, suggesting that their risk for severe outcomes was not increased. BMI and HbA1c in hospitalized vs. non-hospitalized patients were ~24 kg/m^2^ vs. ~25.5 kg/m^2^ and ~8% vs. 7.7%, respectively. It should be noted that the vast majority of patients in this study were using CGMS and 25% of them were on insulin pumps. Research data regarding the effect of COVID-19 infection in a pediatric population with T1DM are scarce. At present, evidence suggests that, contrary to adults, children and adolescents with T1DM infected with SARS-CoV-2 have similar clinical outcomes without increased morbidity and mortality compared to peers without diabetes. A pediatric population with T1DM and COVID-19 infection usually did not require hospitalization [31,32]. Two reports by Rabbone et al. [33] and Unsworth et al. [34] demonstrated that children with type 1 diabetes mellitus and PCR-confirmed SARS-CoV-2 infection presented with mild or no symptoms and did not display long-term adverse outcomes. Regarding the association of COVID-19 infection with the risk of new-onset diabetes, research data are inconsistent. Unsworth et al. in a multicenter study in the UK reported an important increase in new-onset T1DM in children [34]. However, Tittel et al. [35] pointed out that the percentage of new-onset TIDM observed across Germany from March to May 2020 did not differ significantly compared to rates based on data collected over the last decade. In general, several potential factors, alone or in combination, may increase the susceptibility of subjects with T1DM to serious illness following COVID-19 infection.

## 4. The Association of T1DM with SARS-CoV2 Infection: Pathophysiological Mechanisms

**Figure 1:**
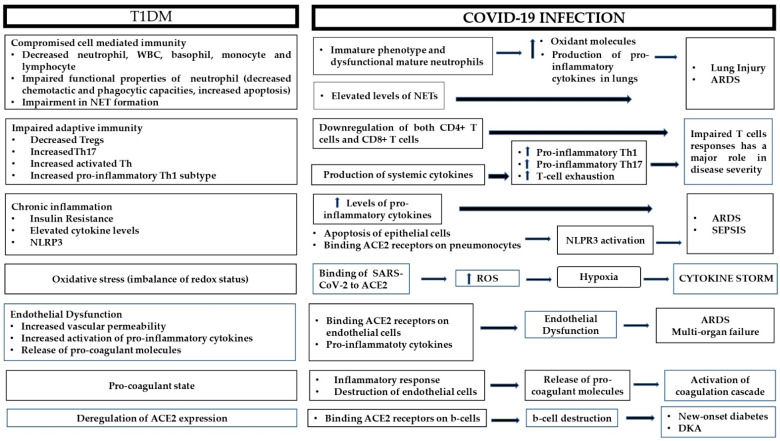
Possible pathophysiological mechanisms responsible for adverse outcomes during COVID-19 infection in patients with type 1 diabetes. ARDS: acute respiratory distress syndrome; ACE2: angiotensin converting enzyme 2; DKA: diabetic ketoacidosis; NET: neutrophil extracellular trap; NLRP3: NOD-like receptor family pyrin domain-containing 3; ROS: reactive oxygen species; Th: T-helper; Tregs: regulatory T-cells; WBC: white blood cells.

The dysregulated metabolic milieu in T1DM exceeds far beyond hyperglycemia; in fact, T1DM is a combination of chronic inflammation and immune dysfunction, which exerts deleterious effects on the vasculature and the coagulation cascade, among others. On the other hand, COVID-19 is a hyperinflammatory condition that ends up as a multi-organ disease. Below, the most prominent connections between the pathophysiology of T1DM and adverse COVID-19 clinical outcomes are presented (Figure 1).

### 4.1. Immune Derangement

Apart from the well-documented role of immune dysregulation in the pathogenesis of T1DM [36], altered immune responses also contribute to the increased susceptibility of diabetic patients to several infections, and presumably SARS-CoV-2. Humoral immunity, which is mediated by macromolecules such as antibodies and complement proteins, does not seem to be seriously aggravated in T1DM; serum antibody titers after vaccination for pneumococcal pneumonia or influenza were similar between diabetic individuals and controls [37,38]. On the contrary, cell-mediated immunity is rather compromised. Despite early data, more recent studies have shown decreased neutrophil numbers in patients with T1DM, a finding that was more prominent at the early stages of the disease [39,40]. Harsunen et al. [41] showed decreased total white blood cells (WBC), neutrophil, basophil, monocyte and lymphocyte counts in 107 adult patients with newly diagnosed type 1 diabetes compared to controls, and the results from Valle et al. [39] were similar, where a mild but significant neutropenia was noted. More importantly, in terms of functional properties, neutrophils have shown decreased chemotactic and phagocytic capacities in T1DM, along with increased rates of apoptosis, with these effects being independent of glycemic control as determined by HbA1c levels [42,43]. Immature phenotype and dysfunctional mature neutrophils have been recognized as pivotal mediators of severe COVID-19 disease by contributing to the acute respiratory distress syndrome (ARDS) pathophysiology through the enhanced production of potent oxidant molecules, such as superoxide radicals and H2O2, and by maintaining the inflammatory state in the lungs by triggering the production of pro-inflammatory cytokines [44,45]. In addition, another important neutrophil-mediated mechanism in the pathogenesis of COVID-19 is NETosis [46]. Neutrophils release granular proteins and chromatin fibers to form an extracellular matrix known as neutrophil extracellular trap (NET), which immobilizes and degrades pathogens such as bacteria and viruses and, therefore, contributes to immune defense [47]. A persistent NET formation, however, exerts a pro-inflammatory effect, as is the case in COVID-19, where elevated levels of NETs have been associated with vascular complications, lung injury and disease severity [46]. In a study by Wang et al. in individuals with T1DM, the circulating levels and enzymatic activities of neutrophil elastase (NE) and proteinase 3 (PR3), which are proteins closely associated with elevated formation of NETs, were dramatically elevated, suggesting a state of sustained chronic inflammation similar to COVID-19 [48]. Contrary to this finding, Qin et al. [49] demonstrated reduced levels of these proteins in 44 T1DM individuals as a result of the lower neutrophil numbers, in accordance with other reports where a delayed and impaired NET formation was noticed as a result of hyperglycemia [50,51]. Even a diminished NETosis, however, could be considered a predisposing factor for adverse COVID-19 outcomes, as any impairment in NET formation is a sign of compromised host defense against pathogens.

Even more importantly than impaired innate immunity, defects in adaptive immunity have also been observed in patients with T1DM. Earlier studies in T1DM patients have reported conflicting results regarding Th17 cell populations [52]; however, more recent data have pointed out an imbalance between Th17 and regulatory T (Treg) cells. In a study by Stanisławowska et al. in children with type 1 diabetes, the analysis of Tregs cells in peripheral blood revealed a lower percentage and the absolute number of CD4+Foxp3+ regulatory T-cells compared to controls; on the contrary, a higher frequency and absolute number of CD4+IL17A+ Th17 cells was demonstrated in the patient group [53]. Monocytes isolated directly from the blood of T1DM patients secreted higher amounts of the pro-inflammatory cytokines IL-6 and IL-1β compared to controls, which are known to induce the expansion of Th17 cell population [54], and a similar effect for IL-1β was demonstrated in peripheral blood mononuclear cells (PBMCs) from children with diabetes [55]. The German Diabetes Study [56] indicated that, in subjects with T1DM, the percentage of activated T-helper and cytotoxic T-cells was higher compared to controls, while the ratio of Treg cells to activated Th cells was lower, implying a defective regulatory capacity, in line with most previous reports [57,58]. Indeed, Treg cell responses might be altered in the course of COVID-19 disease, being increased during the progression to a severe condition and subsequently declining during the progression to a critical condition [59]. A Th1/Th2 imbalance in favor of the pro-inflammatory Th1 subtype with higher activated CD4+CD25+ Th cell subsets has been described in diabetic individuals [60]; however, even in cases where levels of CD4+CD25+ cells are normal, the ability of Tregs to suppress T-cell proliferation was markedly reduced [61]. SARS-CoV-2 infection leads to the downregulation of both CD4+ T-cells and CD8+ T-cells; in addition, the enhanced production of systemic cytokines such as IL-6, IL-1β or CXC Chemokine Ligand 8 (CXCL) leads to skewed T-cell responses, with T-cell exhaustion, hyperactivation of the T helper 1 (Th) subset, and a higher ratio of T helper 17 cells, both of which have a pro-inflammatory effect [60,62]. In general, the imbalance between different T-cell subsets along with impaired T-cell responses in T1DM lead to deficits in adaptive immunity, which could aggravate COVID-19 outcomes.

### 4.2. Chronic Inflammation and Oxidative Stress

Severe, life-threatening COVID-19 disease is characterized by a state of fulminant hypercytokinemia known as “cytokine storm”. Most data about the association between this hyperinflammatory condition and diabetes have been derived from studies on type 2 diabetic patients, as insulin resistance, which is the main factor contributing to the pathogenesis of the disease, has long been considered a state of chronic, low-grade inflammation [21,63]. This mechanism applies also to T1DM to a significant extent as, despite being primarily characterized by insulin deficiency, it is also commonly associated with prominent insulin resistance, which is related mostly to the peripheral tissues rather than the liver [64,65]. Regarding insulin resistance especially at the level of adipose tissue, various studies have shown higher circulating free fatty acid (FFA) and glycerol levels during low-dose hyperinsulinemic and euglycemic clamps, indicating a lower rate of insulin-mediated lipolysis regardless of glycemic control [66]. Increased FFAs activate Toll-like receptor-4 (TLR-4) in adipocytes and macrophages, thus upregulating NF-kB signaling and increasing the production of pro-inflammatory cytokines such as TNF-α [67]. Levels of omentin-1, an adipokine that downregulates the expression of NF-kB and TNF-α and inhibits lipopolysaccharide (LPS)-induced inflammation, are decreased in T1DM [68]; in the same notion, levels of adiponectin, an adipokine that also inhibits TNF-a-induced activation of NF-kB signaling and endothelial adhesion molecule expression, are diminished during hyperglycemic states and are negatively associated with insulin resistance [69]. Studies in mice have shown that the dysfunctional adipose tissue in T1DM results in decreased synthesis of pro-resolving lipid mediators, such as lipoxins, leading to the impaired resolution of chronic inflammation [70]. Even in the absence of insulin resistance, however, chronic inflammation with elevated cytokine levels has been demonstrated in many studies with T1DM subjects. In a study by Gouda et al. [71], serum levels of IL-1α and IL-1β were elevated in T1DM patients in an age-dependent manner, in accordance with the findings by Dogan et al. [72], where elevated levels of IL-1β, IL-2, IL-6, and TNF-a were reported in 27 children with T1DM compared to controls. Regarding IL-6, which is secreted by T-cells and macrophages and plays a crucial role in the pathogenesis of COVID-19 complications, a recent study by Talaat et al. [73] showed higher levels in diabetic children compared to controls, a finding that was confirmed in the studies by Bradshaw et al. [54] and Ururahy et al. [74], which showed higher secretions from monocytes and higher IL-6 mRNA levels in peripheral blood leukocytes in T1DM patients compared to controls, respectively. In a subset of the DCCT/EDIC cohort, where biomarkers were measured at four time points over 20 years in 886 T1DM patients, IL-6, along with tumor necrosis factor receptor-1 and -2, active and total plasminogen activator inhibitor-1 (PAI-1), adhesion molecules and acute-phase reactants, namely fibrinogen and C-reactive protein (CRP), were increased throughout the study period [75]. In patients with T1DM without macrovascular disease, monocyte IL-6 levels were increased compared to controls both in the resting state and after lipopolysaccharide activation, with the latter being the case also for IL-1β [76]. Lastly, regarding TNF-α, the stimulation of PBMCs in T1DM patients with high-M alginate and lipopolysaccharides (LPS) induced a significantly increased TNF production compared to controls [77], and Hussain et al. [78] showed a substantial hypersecretion of IL-1α and TNF-α from PBMCs both in 29 T1DM children and their healthy first-degree relatives. More importantly, in a case–control study from the EURODIAB Prospective Complications Study of 543 individuals with T1DM, plasma levels of CRP, IL-6, TNF-α, VCAM, and E-selectin were significantly higher in patients with macrovascular complications compared to those without [79], suggesting the role of chronic inflammation in the pathogenesis of diabetic micro- and macrovascular complications, which, in turn, have been considered the major risk factor of adverse COVID-19 outcomes in many cohorts. In general, the natural history of COVID-19 encompasses excessive circulating levels of pro-inflammatory cytokines such as IL-1β, IL-6, IL-7, IL-8, IL-17, interferon gamma (IFNγ), TNF-α, CXCL10 and monocyte chemoattractant protein-1 (MCP-1), and is associated with progression to ARDS, sepsis and acute mortality [80,81]. In fact, the presence of increased levels of IL-1β in patients with COVID-19 infection revealed that the inflammatory process is preceded by cell pyroptosis, which is mediated through the activation of the NOD-like receptor family pyrin domain-containing 3 (NLRP3) inflammasome [67]. T1DM-related oxidative stress, mediated through the increased production of ROS and AGEs, induces the formation of the NLRP3 inflammasome through the activation of the NF-kB pathway [82]. At the same time, SARS-CoV-2 activates the inflammasome through various mechanisms. More specifically, as SARS-CoV-2 enters the alveolar epithelial cells, it leads to their apoptosis, which, in turn, leads to the release of molecules that trigger NRP3 activation of the alveolar macrophages [83]. The virus also activates the inflammasome by binding ACE2 receptors on pneumonocytes, while its N proteins activate the complement cascade and release the C3a and C5a anaphylatoxins, which induce inflammasome activation as well [84].

**Figure 2:**
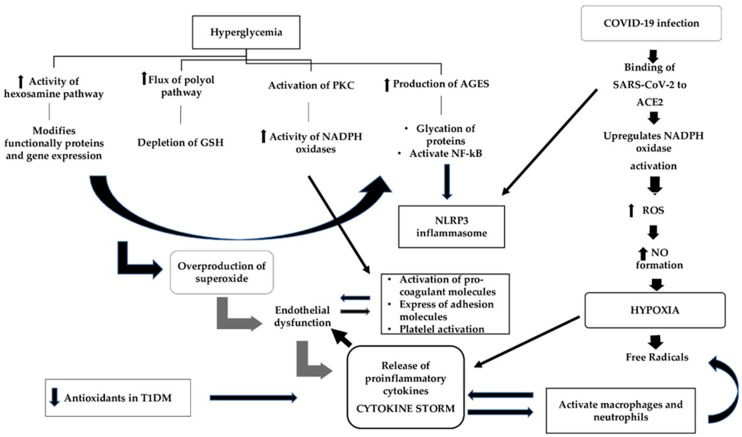
The mechanisms of oxidative stress leading to adverse clinical outcomes during COVID-19 infection in patients with type 1 diabetes mellitus. In T1DM, hyperglycemia is a potent mediator of oxidative stress through four different pathways whose accumulative effect is the overproduction of superoxide, which leads to endothelial dysfunction and the subsequent release of pro-inflammatory cytokines. The destroyed endothelial cells release pro-coagulant molecules and express adhesion molecules. Simultaneously, hyperglycemia through the activation of the PKC pathway increases the expression of adhesion molecules, contributing additionally to endothelial dysfunction, and this leads to platelet activation and aggregation. During COVID-19 infection, the binding of SARS-CoV-2 to angiotensin-converting enzyme 2 (ACE2) results in the increased production of reactive oxygen species (ROS), which in turn increases NO formation and leads to cytopathic hypoxia. Hypoxia via the formation of free radicals upregulates the release of pro-inflammatory cytokines, which promotes endothelial derangement and contributes to pro-coagulability. Additionally, the upregulation of pro-inflammatory cytokines, in a vicious cycle, activate macrophages and neutrophils to produce more free radicals. Neutrophils have been recognized as pivotal mediators of severe COVID-19 disease by triggering the production of pro-inflammatory cytokines and maintaining the inflammatory state in the lungs. AGEs: advanced glycation end products; GSH: glutathione; NADPH: nicotinamide adenine dinucleotide phosphate; NF-kB: nuclear factor-kB; NLRP3: NOD-like receptor family pyrin domain-containing 3; NO: nitric oxide; PKC: protein kinase C; ROS: reactive oxygen species.

Apart from inflammation, oxidative stress is a key player in COVID-19; in fact, it is the interplay between these two pathways that determines disease severity [85] (Figure 2). Oxidative stress is a state of imbalance between the production of ROS and antioxidant defenses that inactivates these free radicals [86]. ROS includes superoxide radicals (O_2_^•−^), hydrogen peroxide (H_2_O_2_), hydroxyl radicals (^•^OH), and singlet oxygen (^1^O_2_), all necessary by-products of mainly mitochondrial metabolism that do become harmful when accumulating at high levels. To defend themselves from the deleterious effects of these mediators, cells produce enzymatic antioxidants including mainly superoxide dismutase (SOD), catalase (CAT), and glutathione peroxidase (GPx), which delay or even utterly inhibit the oxidization process [87]. Hyperglycemia, the prominent feature of T1DM, has long been known to be a potent mediator of oxidative stress through four distinct pathways [88]: (a) the increased flux of the polyol pathway, which leads to the depletion of glutathione (GSH), a potent antioxidant, (b) the activation of protein kinase C (PKC), which leads to increased activity of NADPH oxidases, (c) the increased intracellular production of advanced glycation end products (AGEs), which leads to the glycation of certain proteins that bind to AGE receptors (RAGE) and activate nuclear factor kappa B (NF-kB) and other inflammatory pathways, and (d) the increased activity of the hexosamine pathway, leading to the production of UDP-N-acetyl glucosamine, which modifies functionally important proteins and, eventually, gene expression. The cumulative effect of these pathways is the overproduction of superoxide from the mitochondria, which promotes endothelial dysfunction and the subsequent release of pro-inflammatory cytokines and adhesion molecules from the damaged cells [89]. Many studies have demonstrated the presence of oxidative stress markers in patients with T1DM. In a study by Fatima et al. [90], the activity of antioxidant enzymes in plasma such as superoxide dismutase, catalase, and glutathione reductase was significantly downregulated in 29 T1DM subjects compared to healthy controls, with a concomitant increase in the levels of pro-inflammatory cytokines IL-1β, IL-17A, IL-23, IFN-γ and TNF-α. Malondialdehyde (MDA) [91] and protein carbonyl (PC) [92], which are reliable biomarkers of oxidative stress, were found to be elevated in the plasma of T1DM patients, indicating enhanced polyunsaturated fatty acid peroxidation and free radical-mediated protein damage. In a study including 35 patients with T1DM and 28 age- and sex-matched normal subjects, MDA level was significantly elevated in diabetic patients, while the level of GSH and the ferric reducing ability of plasma (FRAP) were lower in patients than in controls, and these findings were associated with poorer glycemic control [91]. This finding about GSH has been observed in a number of studies showing decreased intracellular glutathione concentrations in both the plasma and erythrocytes of patients with T1DM [93,94], and the same applies to other antioxidant molecules such as catalase (CAT) [95] and superoxide dismutase (SOD) [96]. The imbalance of redox status that is observed in T1DM [97] leads to altered immune responses and increased levels of proinflammatory cytokines that are key constituents of the “cytokine storm”, such as IL-1β. During COVID-19 infection, the high affinity and the consequent binding of SARS-CoV-2 to angiotensin-converting enzyme 2 (ACE2) increases angiotensin II availability and upregulates nicotinamide adenine dinucleotide phosphate (NADPH) oxidase (NOX) activation, which leads to the increased production of reactive oxygen species (ROS) [98,99]. ROS, in turn, increases iNOS expression via NF-κB activation and, therefore, increases NO formation, which results in cytopathic hypoxia [100]. This hypoxia, which is a common manifestation of sepsis, triggers the formation of free radicals through the mitochondrial respiratory chain, an event that eventually leads to the upregulation of pro-inflammatory cytokines that, in a vicious cycle, activate macrophages and neutrophils to produce more free radicals [101]. Oxidative stress converts soluble plasma fibrinogen into abnormal fibrin clots, resulting in microthrombosis, while the attack of SARS-CoV-2 against hemoglobin groups in the erythrocyte leads to the release of heme and free iron ions into the bloodstream, further contributing to ROS production and mitochondrial damage [102].

### 4.3. Endothelial Dysfunction

The endothelium plays a vital role in several physiological processes; it regulates the tone of vascular smooth muscle by the release of vasodilators and vasoconstrictors, controls vascular permeability, and is an important mediator of inflammation and coagulation by regulating the adhesion of leucocytes and platelets [103,104,105]. The endothelial glycocalyx is an endothelial surface layer that consists of proteoglycans and glycoproteins and prevents the direct contact of blood cells with the endothelial surface [106].

According to several research data, T1DM is associated with endothelial dysfunction [107,108,109]. A cross-sectional study showed that endothelial function assessed by flow-mediated dilation (FMD) is impaired in patients with T1DM compared to healthy individuals, even in patients with disease duration less than five years [107]. Jarvisalo et al. reported that 16 (36%) out of 45 patients with T1DM displayed lower peak FMD response and increased carotid intima-mediated thickness, suggesting that endothelial dysfunction may be an indicator for the development of premature atherosclerosis [108]. Another study by Machnica et al. reported that markers of endothelial destruction, including intercellular adhesion molecule-1 (ICAM-1), VCAM-1, E-selectin, TNF-a and IL-6, were significantly increased in patients with T1DM and disease duration 5.13 ± 2.18 years compared to controls, indicating that endothelial dysfunction is present early in the course of T1DM [109]. Endothelial dysfunction and endothelial glycocalyx damage increase leucocyte and circulating inflammatory cells’ adhesion, and promote vascular permeability and coagulation [110,111,112,113]. The pathogenesis of the impairment of the endothelial micromilieu in patients with T1DM is closely related to hyperglycemia and subsequent oxidative stress [70]. The passive diffusion of glucose into the endothelial cells and the subsequent intracellular accumulation of glucose during acute and long-term hyperglycemia activates sorvitol, protein kinase C, and pentose phosphate pathways, and increases the NADPH/nicotinamide adenine dinucleotide (NAD) rate. These alterations reduce the availability of nitric acid, increase vascular permeability, promote an inflammatory response via the activation of cytokines and adhesion molecules, and contribute to endothelial cells’ apoptosis and endothelial dysfunction [70,114,115]. The destroyed endothelial cells release pro-coagulant molecules (von Willebrand factor, PAI-1 and tromboxan A2) and express adhesion molecules including P-selectin, E-selectin, VCAM-1, and ICAM-1, which contribute to the adhesion of neutrophils and platelets to endothelium [116]. Hyperglycemia and the subsequent production of advanced-glycation end products are associated with the increased expression of pro-inflammatory cytokines including IL-6, TNF-α and IL-1, which enhance the expression of pro-coagulant molecules and inhibit the release of anti-coagulant molecules (thrombomodulin) by endothelial cells [117,118,119]. Consequently, endothelial derangement promotes a pro-inflammatory and a pro-coagulant state.

SARS-CoV-2 affects the vascular system directly by targeting endothelial cells through ACE2 receptor binding, leading to severe endothelial derangement and inflammation [120,121,122]. In addition, the overproduction of pro-inflammatory cytokines during COVID-19 promotes endothelial dysfunction [83]. Chen et al. demonstrated that patients with severe COVID-19 disease displayed increased levels of pro-inflammatory cytokines, including soluble interleukin 2 receptor (IL-2R), IL-6, and TNF-a [7]. IL-6 promotes endothelial derangement and contributes to pro-coagulability [123,124]. Furthermore, the inflammatory-mediated damage of glycocalyx by IL-6 or TNF-α increases vascular permeability inducing interstitial fluid shift and generalized edema [125].

The infected endothelial cells release increased levels of proinflammatory cytokines, which induce the immune-mediated damage of lungs and other organs resulting in ARDS and multi-organ failure [126]. Histological studies showed that endothelial cells damaged by SARS-CoV-2 result in vasculitis and endothelitis in multiple organs [127,128,129]. Postmortem histological reports from three patients gave the first evidence of the role of endothelium in the pathogenesis of COVID-19 disease, and provided evidence of endothelitis during the course of the infection. The authors suggested that the clinical presentation of COVID-19 might be worse in vulnerable patients with pre-existing endothelial dysfunction [127]. Copin et al. showed that endothelial derangement induces the vascular damage of small to medium-sized pulmonary arteries, contributing to lung injury in severe COVID-19 disease [129].

A chronic endothelial dysfunction predisposes one to severe COVID-19 infection by inducing alterations at the glycocalyx and endothelial cells, leading to increased leucocyte adhesion and promoting a procoagulant and antifibrinolytic state. Chronic endothelial dysfunction due to T1DM in combination with the direct damage of endothelial cells by SARS-CoV-2 may result in endothelial and microcirculation impairment, which contribute to the pathogenesis of acute respiratory syndrome and multi-organ failure.

### 4.4. Coagulopathy

Type 1 diabetes mellitus is associated with a hypercoagulant state, which is characterized by the deregulation of clotting and fibrinolytic activity. One responsible mechanism for these derangements is the alteration in the concentration and activity of several coagulatory proteins, mainly due to hyperglycemia [130]. According to research data, several pro-coagulant proteins, including von Willebrand factor, prothrombin, and fibrinogen, are increased in T1DM [131,132,133,134]. Additionally, plasmin presents reduced activity due to the increased glycation of plasminogen, the precursor of plasmin [135]. Simultaneously, the concentrations of anti-coagulant proteins, including protein C and protein S, are reduced [136,137]. The activity of antithrombin is also impaired by methylglyoxal, a by-product of hyperglycemia [138]. Regarding the anti-fibrinolytic proteins, the concentrations of thrombin-activatable fibrinolysis inhibitor and 2-macroglobulin are elevated in patients with T1DM [139,140]. The aforementioned derangements in coagulatory proteins concentrations and activity exert a negative impact on fibrin clot formation and clot lysis parameters, resulting in a pro-coagulant state that increases the risk of thrombosis [130]. Another mechanism responsible for the hypercoagulability state in T1DM is the hyperactivation of platelets. Hyperglycemia induces the reduction of nitric acid and prostacyclin production by endothelial cells, leading to an imbalance in platelet activity inhibition [141]. Additionally, hyperglycemia and the subsequent increase in oxidative stress activates the PKC pathway, which contributes to platelet activation and aggregation [142,143]. The glycation of proteins at the surface of platelets ameliorates the sensitivity of platelets to thrombin and increases platelets adhesion [144]. Consequently, hyperglycemia is the main mediator which, through the imbalance of coagulatory proteins and the hyperactivation of platelets, promotes a hypercoagulability state in T1DM.

A significant quantity of research data regarding COVID-19 patients showed that SARS-CoV-2 infection is associated with hypercoagulation in multiple organs, as denoted by increased levels of D-dimers and fibrinogen degradation products [145,146]. Zhou et al. reported that 117 (68%) out of 172 patients with COVID-19 infection presented increased activation of coagulation, as indicated by high levels of D-dimer concentrations. Interestingly, D-dimer concentrations above 1 μg/mL were associated with an 18 times increased odds ratio for a fatal outcome [145]. Α study by Klok et al. with 184 ICU patients pointed out that 31% of the patients presented thrombotic complications including venous thromboembolism (27%) and arterial embolism (3%), despite systematic thrombosis prophylaxis [147]. A retrospective study from China including 183 patients highlighted that 71% of patients met clinical criteria for disseminated intravascular coagulation (DIC), and that impaired coagulation parameters (D-dimers, fibrin degradation product, prothrombin time, fibrinogen and antithrombin activity) are significantly associated with prognosis [148]. An autopsy study in skin and lung tissues from five individuals with severe COVID-19 infection identified a generalized microvascular thrombotic injury [149]. Profound inflammatory response and the activation of cytokine storm in COVID-19 infection, in conjunction with the destruction of the endothelial cells with the subsequent release of pro-coagulant molecules, contribute to the activation of the coagulation cascade.

### 4.5. The Role of ACE2

Angiotensin-converting enzyme (ACE) is the key mediator of the renin angiotensin aldosterone system (RAAS) by converting angiotensin I to II. ACE2 is a novel homolog of ACE that degrades mainly angiotensin II, and to a lesser extent angiotensin I to angiotensin I-VII and angiotensin I-IX [150,151]. According to research data, angiotensin I-VII ameliorates vasodilation, stimulates bradykinin release, inhibits ACE, and subsequently diminishes vasoconstriction mediated by angiotensin II. Thus, angiotensin I-VII seems to be an important modulator of the RAAS [152,153]. The decreased ACE2 expression results in increasing vascular permeability by the activation of the kallikrein–bradykinin pathway [154]. Additionally, ACE2 and angiotensin I-VII physiologically seem to display important anti-inflammatory and anti-oxidant properties [155]. The reduction in ACE2 expression leads to cellular damage and hyperinflammation. 

ACE2 is expressed in various human tissues, including lungs, hearts, kidneys, intestine and blood cells. Angiotensin-converting enzyme 2 (ACE2) receptors have been identified as the main target of the SARS-CoV-2 spike protein, permitting the entrance of the virus in host cells. [120,151]. According to research data, chronic hyperglycemia leads to the downregulation of ACE2 expression, rendering the host cells susceptible to the inflammatory and damaging effect of the SARS-CoV-2 [156]. On the other hand, acute hyperglycemia upregulates ACE2 expression, which enables increased virus entry into the cells [157]. A study with mouse models of diabetes showed that the expression of ACE2 receptors is increased in multiple tissues [158]. This deregulation of ACE2 expression results in both an increased risk of COVID-19 infection and adverse outcomes of the disease. ACE2 is also expressed in pancreatic b-cells, which may permit the direct damage of b-cell function [159]. A recent study by Mȕller et al. showed that SARS-CoV-2 infects and replicates in cultured human islets, inducing morphological and functional abnormalities such as impairment in glucose responsiveness [160]. Similarly, another study highlighted that SARS-CoV-2-associated b-cell infection leads to inflammatory cytokine release, b-cell apoptosis and decreased insulin secretion [161]. Type 1 diabetes results from auto-immune destruction and primary losses of b-cell mass; however, residual b-cell function is retained in many individuals for decades after clinical diagnosis, contributing to glycemic control [162]. The binding of SARS-CoV-2 to ACE2 receptors of b-cells may lead to the destruction of the remaining b-cells and to subsequent complete insulin deficiency, which contributes to the development of severe ketoacidosis. Indeed, Li et al. reported that 6.4% of patients with COVID-19 and 11.6% of patients with COVID-19 and diabetes presented ketosis, which resulted in a high mortality rate (33.3%). The authors concluded that COVID-19 infection leads to the development of ketosis or DKA in patients without diabetes, and induces DKA in patients with diabetes [163]. Τhe Italian Society of Pediatric Endocrinology and Diabetes reported that the percentage of children with severe DKA rose from 36.1% to 44.3% during the early phase of COVID-19 [33]. Similarly, a multicenter study from Germany reported that the incidence of DKA in 532 children and adolescents with T1DM was 44.7% during the COVID-19 period in 2020, which was significantly higher compared to the two previous years [164]. Οn the other hand, the direct damage of pancreatic cells by SARS-CoV-2 may contribute to the development of new-onset diabetes. A recent case report presented a 19-year-old German male who was hospitalized with DKA and insulin-dependent diabetes in the absence of typical type 1 diabetes autoantibodies, 5 to 7 weeks after asymptomatic COVID-19 infection [165]. Another case report described a patient who presented DKA and was diagnosed with T1DM at the onset of COVID-19 infection [166]. A multicenter study in the UK was the first that reported an important increase in new-onset T1DM in children, with evidence of SARS-CoV-2 infection or exposure in some of these [34]. Research data regarding the effect of other coronavirus outbreaks showed that SARS-associated b-cell damage causes acute hyperglycemia, even among people without diabetes. Approximately 50% of SARS patients who had no previous diabetes and received no steroid treatment developed diabetes during hospitalization, and after 3 years, 5% of the patients still had diabetes. These findings support the probability of chronic damage to b-cells [167].

## 5. Conclusions

A growing number of studies have demonstrated that T1DM is an important risk factor affecting the clinical severity of COVID-19 disease. Immune and inflammatory dysregulation in conjunction with increased oxidative stress render patients with T1DM susceptible to severe COVID-19 infection. Additionally, the chronic endothelial dysfunction and the hypercoagulant state observed in T1DM account for the generalized endothelitis and thrombotic events that culminate in multi-organ failure and death. The deregulation of ACE2 expression in T1DM results in both increased risk of COVID-19 infection and in adverse outcomes of the disease. Apart from its role in viral transmission, the ACE2 receptor could also contribute to the development of new-onset diabetes and the induction of DKA in patients with T1DM. Large clinical studies are required to identify the clinical and biochemical parameters that could help identify patients at greater risk so that, along with a better understanding of the underlying pathophysiological mechanisms, more precise, timely and individualized therapeutic decisions can be made.

## Abbreviations

ACE2	angiotensin-converting enzyme 2
AGEs	advanced glycation end products
ARDS	acute respiratory distress syndrome
BMI	body mass index
CAT	catalase
CGMS	continuous glucose monitoring systems
COVID-19	coronavirus disease
CRP	C-reactive protein
CXCL	CXC chemokine ligand
DIC	disseminated intravascular coagulation
DKA	diabetic ketoacidosis
FMD	flow-mediated dilation
FRAP	ferric reducing ability of plasma
GSH	glutathione
ICAM-1	intracellular adhesion molecule 1
IFN-γ	interferon gamma
IL	interleukin
IR	insulin resistance
LPS	lipopolysaccharide
MCP-1	monocyte chemoattractant protein-1
MDA	malondialdehyde
NAD	nicotinamide adenine dinucleotide
NADPH	nicotinamide adenine dinucleotide phosphate
NE	neutrophil elastase
NET	neutrophil extracellular trap
NF-kB	nuclear factor-kB
NLRP3	NOD-like receptor family pyrin domain-containing 3
NO	nitric oxide
NOX	oxidase
OS	oxidative stress
PAI-1	plasminogen activator inhibitor-1
PBMC	peripheral blood mononuclear cells
PC	protein carbonyl
PKC	protein kinase C
PR3	proteinase 3
RAAS	renin angiotensin aldosterone system
RAGE	advanced glycation end products receptors
ROS	reactive oxygen species
SARS	severe acute respiratory syndrome
SARS-CoV-2	severe acute respiratory syndrome coronavirus 2
SOD	superoxide dismutase
T1DM	type 1 diabetes mellitus
T2DM	type 2 diabetes mellitus
Th	T-helper
TNF-α	tumor necrosis factor alpha
Treg	T regulatory
VCAM	vascular cell adhesion molecule
WBC	white blood cells
WHO	World Health Organization
